# 

**Published:** 1913-08-09

**Authors:** 


### NEW OFFICERS OF THE ROYAL COLLEGE OF PHYSICIANS.

At the ordinary quarterly *Comitia* of the Royal College of Physicians held last week the following Fellows of the college were appointed officers for the ensuing collegiate year: Censors, Sir J. Kingston Fowler, K.C.V.O., M.D., W. Hale White, M.D., Howard H. Tooth, C.M.G., M.D., T. Dyke Acland, M.D.; Treasurer, Sir Dyce Duckworth; Emeritus Registrar, Edward Liveing, M.D.; Registrar, J. A. Ormerod, M.D.; Harveian Librarian, Norman Moore, M.D.; Library Committee, H. D. Rolleston, M.D., L. G. Guthrie, M.D., H. M. Fletcher, M.D., R. H. P. Crawfurd, M.D.; Curators of the Museum, J. M. Bruce, M.D., S. J. Sharkey, M.D., F. W. Andrewes, M.D., and W. Hunter, M.D.; Finance Committee, Dr. Donald Hood, C.V.O., M.D., H. W. G. Mackenzie, M.D., and Sir James Reid, K.C.V.O., M.D.

### THE RAYMOND HORTON-SMITH PRIZE.

The Raymond Horton-Smith Prize for 1913 is awarded to F. A. Roper, of Trinity College, and F. S. Scales, of Jesus College, who are adjudged equal for theses for the degree of Doctor of Medicine. The subjects were: "Creatinine and Creatin Metabolism, especially in Reference to Diabetes," and "The Electrocardiogram as an Aid to Diagnosis." The M.D. Degree Committee desire to place on record their appreciation of the high standard attained by most of the theses submitted for the degree of Doctor of Medicine. Among the theses not eligible to compete for the Raymond Horton-Smith Prize that submitted by W. E. Hume, M.A., M.D., of Pembroke College, on "A Clinical and Pathological Study of the Heart in Diphtheria" attained a very high standard of merit.

### THE LATE SIR J. W. SCOTT.

A generous contributor to the Bolton Infirmary has passed away with the death of Sir James William Scott, aged sixty-nine. His wealth was made chiefly in the cotton trade. Always a keen supporter of charity, he helped to found the Guild of Help, and was president of the oldest charity in Bolton—the Poor Protection Society. He used to entertain crippled children at his Bolton residence every year, and was a strong supporter of Bolton Infirmary.

### Appointments.

The following house appointments have been made at St. Thomas's Hospital from August 5 and 19, 1913, subject to the approval of the Treasurer:—Casualty officers and resident anæsthetists (to begin on August 19), W. J. Hart, B.M., B.Ch.(Oxon.), M.R.C.S., L.R.C.P.; M. J. Petty, M.R.C.S., L.R.C.P.; F. McG. Loughnane, M.R.C.S., L.R.C.P.; E. N. Butler, M.R.C.S., L.R.C.P.; A. L. Sutcliffe, M.B., B.C., M.R.C.S., L.R.C.P.; H. C. Attwood, M.R.C.S., L.R.C.P.; W. B. Foley, M.B., B.S., M.R.C.S., L.R.C.P.; and A. Viney, M.R.C.S., L.R.C.P. Casualty assistants (to begin on August 5), E. L. K. Sargent, M.B., B.C., M.R.C.S., L.R.C.P., and L. G. Bourdillon, M.R.C.S., L.R.C.P. Resident house physicians (to begin on August 5), H. P. Dawson, M.R.C.S., L.R.C.P.; E. Wordley, M.B., B.C., M.R.C.S., L.R.C.P.; S. G. Askey, M.R.C.S., L.R.C.P.; and D. B. I. Hallett, M.B., B.Ch.(Oxon.), M.R.C.S., L.R.C.P. Resident house surgeons (to begin on August 5), F. E. Daunt, M.B., B.S.(Lond.), M.R.C.S., L.R.C.P.; W. J. F. Symons, M.B., B.C., M.R.C.S., L.R.C.P.; and P. G. Doyne, M.R.C.S., L.R.C.P.; and L. N. Reece, M.R.C.S., L.R.C.P. House surgeon to Block 8 (to begin on August 19), P. H. Mitchiner, M.B., B.S.(Lond.), L.R.C.P.(Lond.), F.R.C.S.(Eng.). Obstetric house physicians (to begin on August 19), A. R. Chavasse, B.M., B.Ch.(Oxon.), M.R.C.S., L.R.C.P., and F. J. Humphrys, M.B., B.S.(Lond.), M.R.C.S., L.R.C.P. Ophthalmic house surgeons (to begin on August 19), G. N. Brandon, M.R.C.S., L.R.C.P.

Dr. E. W. Lowry and Dr. F. W. James, respectively late chairman and secretary of the Ealing Division of the British Medical Association, were presented at Acton Cottage Hospital last week with silver salvers. The donors were "some members of the medical profession resident in the Ealing Division of the British Medical Association, in appreciation of their strenuous work during the crisis of 1913." Dr. Bott presided over the ceremony, and remarked that what began like a rout had ended in defeat with honour, which was due to the work of Dr. Lowry and Dr. James. In reply, Dr. Lowry said that when the next fight arrived he hoped that the lessons which this failure had taught them would lead them to success.

